# Isorhamnetin Modulates Drug-Resistance-Related Biomarkers in Colon Cancer Cells

**DOI:** 10.3390/ijms26136208

**Published:** 2025-06-27

**Authors:** Nikola Radenković, Dejan Milenković, Danijela Nikodijević, Sofija Jovanović Stojanov, Ana Podolski Renić, Milena Milutinović

**Affiliations:** 1 Department of Biology and Ecology, Faculty of Science, University of Kragujevac, Radoja Domanovića 12, 34000 Kragujevac, Serbia; nikolaradenkovic994@gmail.com (N.R.); danijela.nikodijevic@pmf.kg.ac.rs (D.N.); 2 Department of Natural Sciences, Institute for Information Technologies Kragujevac, University of Kragujevac, Jovana Cvijića bb, 34000 Kragujevac, Serbia; 3 Department of Neurobiology, Institute for Biological Research “Siniša Stanković”, University of Belgrade, Bulevar Despota Stefana 142, 11060 Belgrade, Serbia

**Keywords:** colon cancer, multidrug resistance, ATP-binding cassette transporters, P-glycoprotein, multidrug resistance-associated protein 1, multidrug resistance-associated protein 5

## Abstract

The development of resistance to standard cytostatics, such as 5-fluorouracil (5-FU), significantly limits the efficacy of colon cancer therapy, prompting the search for novel anticancer agents, particularly among natural compounds. This study evaluated the anticancer effects of isorhamnetin, a plant-derived flavonol, and its ability to modulate the expression of drug-resistance-related biomarkers in SW-480 and HT-29 colon cancer cells, with a focus on ATP-binding cassette (ABC) transporters. Isorhamnetin demonstrated strong cytotoxic and proapoptotic activity on both cell lines, while showing lower toxicity toward normal HaCaT cells. In addition to suppressing the mRNA expression of drug-metabolizing enzymes (CYP1A1 and CYP1B1), isorhamnetin significantly reduced the mRNA levels of multidrug resistance-associated proteins 1 and 5 (MRP1 and MRP5), as well as the P-glycoprotein (P-gp) level in SW-480 and HT-29 cells. Molecular docking analysis revealed a high binding affinity of isorhamnetin to CYP1A1, CYP1B1, P-gp, MRP1, MRP5, and glutathione S-transferase (GST) proteins, with stronger interactions than those observed for 5-FU, suggesting potential interference with their function. These results provide a solid basis for future investigations to confirm the therapeutic potential of isorhamnetin as a modulator of drug resistance in colon cancer cells.

## 1. Introduction

Data from the Global Cancer Observatory (GCO), as visualized in Cancer Today, report that colon cancer ranks third in incidence and second in mortality among all cancers in 2022 [1]. Estimates, disseminated by Cancer Tomorrow, show that by 2045, the number of new cases will increase by 70.5% and the number of deaths by 83.4% [2].

Although various strategies have been developed for the treatment of colon cancer, chemotherapy remains the primary therapeutic approach. Since the mid-20th century, 5-fluorouracil (5-FU) has been the main cytostatic used in colon cancer therapy [3]. However, the major limitation of this treatment is the development of 5-FU resistance in colon cancer cells, which severely reduces the drug’s effectiveness [4]. Typically, drug resistance arises through several mechanisms, including drug inactivation by drug-metabolizing enzymes. In phase I biotransformation, xenobiotics undergo oxidation, reduction, or hydrolysis reactions mediated by cytochrome P450 (CYP) enzymes. During phase II, xenobiotics are conjugated by glutathione S-transferases (GSTs), primarily with glutathione [5]. Alterations in CYP enzyme activity can result in modified xenobiotic metabolism, leading to enhanced degradation and excretion, while elevated glutathione levels and increased GST activity promote the elimination of xenobiotics from the cells. Additionally, one of the key mechanisms of drug resistance involves an increased efflux of cytostatics and other drugs by transmembrane ATP-binding cassette (ABC) transporters [6,7,8].

The ABC transporters have a broad substrate spectrum that includes numerous cytostatics in use [9]. For this reason, ABC transporters play a significant role in the development of drug resistance. However, only three ABC transporters have been extensively studied concerning drug resistance. These are P-glycoprotein (P-gp; the product of the *MDR1* gene), multidrug resistance-associated protein 1 (MRP1), and breast cancer resistance protein (BCRP) [6,7,8]. In addition, multidrug resistance-associated protein 5 (MRP5) may play an important role in the development of drug resistance in colon cancer cells, as it can efflux cyclic nucleotides and nucleotide analogs [10], including active 5-FU monophosphate metabolites [11]. Although there are some structural differences, most ABC transporters contain transmembrane domains (TMDs) that have hydrophilic nucleotide-binding domains (NBDs) attached. The TMDs are responsible for binding the substrate, while NBDs function as ATPases that hydrolyze ATP, thus providing energy for the translocation of substrates across a membrane [12].

Therefore, one approach to overcoming drug resistance is to identify new compounds that inhibit P-gp, MRP1, BCRP, and other ABC transporters. So far, numerous compounds have been positively tested as P-gp modulators, classified into three generations [9]. The best-known inhibitor of the first generation is verapamil, a calcium channel antagonist. Despite its beneficial properties, verapamil exhibited non-selective and toxic effects due to the high concentration needed to inhibit P-gp [13]. The second generation of inhibitors includes dexverapamil, valspodar, and biricodar. These compounds are more potent and less toxic than those of the first generation. However, several issues have limited their clinical use, such as insufficient specificity for P-gp and interactions with CYP enzymes. Third-generation inhibitors (dofequidar, zosuquidar, tariquidar, and elacridar) were designed to have high specificity for P-gp, but they have not been successful in clinical trials due to their toxicity, potentially resulting from the inhibition of P-gp in normal tissues [14].

Given the limited capacity of the strategies developed so far to overcome drug resistance, the pursuit of new active substances remains relevant. The compounds that have recently garnered significant attention as potential modulators of P-gp and other ABC transporters are natural products known as fourth-generation inhibitors [15,16]. Among these, phytochemicals demonstrate greater potential to succeed than previously developed inhibitors due to their multiple targets, low toxicity, and additional beneficial effects [17,18]. Some of these compounds also exhibit synergistic effects with cytostatics and mitigate resistance by modulating P-gp transport [19]. In this context, intensive research is underway to identify the most appropriate compounds to reverse drug resistance mediated by ABC transporters.

Isorhamnetin (3′-methoxy-3,4′,5,7-tetrahydroxyflavone) is a flavonol aglycone that belongs to the flavonoid class and is widely found in fruits and vegetables as well as in plants used in traditional medicine for the treatment of rheumatism, hemorrhage, and cardiovascular diseases, such as *Hippophae rhamnoides* L. and *Ginkgo biloba* L. [20]. This compound is a metabolite of quercetin, so it is also known as 3′-methylquercetin. Isorhamnetin has been reported to exhibit various pharmacological properties due to its anti-inflammatory, antioxidant, antiosteoporosis, antibacterial, antiviral effects, etc. [21]. As for its anticancer effects, numerous studies have shown that isorhamnetin inhibits the proliferation of colon [22,23], breast [24], bladder [25], liver [26], and skin [27] cancer cells, mainly by inducing apoptosis. Prior investigations have suggested the potential of isorhamnetin to modulate drug resistance mechanisms through interactions with ABC transporters. Our previous study [28] demonstrated that isorhamnetin can modulate the expression of several drug-resistance-associated ABC transporters, including P-gp in DLD-1 and HCT-116 colon cancer cells, thereby influencing cellular drug efflux and resistance development. Other authors reported that isorhamnetin is a substrate of P-gp and can stimulate its ATPase activity in breast cancer cells [29]. Furthermore, Lan and associates [30] demonstrated that isorhamnetin undergoes P-gp-mediated efflux and may enhance the bioavailability of co-administered flavonols, with possible involvement of other ABC transporters such as MRP2 and BCRP. While these studies have provided valuable insights into isorhamnetin’s interactions with ABC transporters, further research is needed to evaluate its effects on a broader spectrum of colon cancer cell lines. In addition, molecular docking approaches could clarify the detailed interactions between isorhamnetin and ABC transporters as well as CYP enzymes, thereby enhancing the mechanistic understanding of their role in modulating drug efflux and metabolism.

Therefore, the present study aimed to investigate the modulatory effects of isorhamnetin on the mRNA expression of drug-resistance-related biomarkers, focusing on various ABC transporters in SW-480 and HT-29 colon cancer cells. To complement these findings, molecular docking analyses were also conducted to characterize the binding affinity of isorhamnetin to target CYP enzymes and ABC transporters, providing mechanistic insights into their potential molecular interactions.

## 2. Results

### 2.1. Anticancer Potential of Isorhamnetin: Cytotoxicity and Proapoptotic Activity

#### 2.1.1. Cytotoxic Effect of Isorhamnetin

The results showed a decrease in the viability of the SW-480 and HT-29 cells (Figure S1) and notable cytotoxicity shown as IC50 (the concentration that inhibits 50% of cell growth) values (Table 1). Although SW-480 cells were highly sensitive to isorhamnetin, time-dependent cytotoxicity was observed only on HT-29 cells.

In addition, we monitored the cytotoxic selectivity (selectivity index (SI) value) of isorhamnetin on normal HaCaT cells (human keratinocytes) (Table 1). The results indicate that isorhamnetin showed a selective effect on SW-480 cells, both after 24 and 72 h. Although it was slightly more cytotoxic to HaCaT cells than HT-29 cells after 24 h, the SI value observed after 72 h indicates that this compound showed a selective effect on HT-29 cells.

Although the IC50 concentration was usually used for evaluation of cytotoxicity and comparison with other anticancer drugs, for further analyses of isorhamnetin effects, we used some less cytotoxic doses, IC25 concentrations (values for 24 h, 0.8 ± 0.04 and 13.04 ± 0.17 µg/mL, for SW-480 and HT-29 cells, respectively).

#### 2.1.2. Isorhamnetin Exerts Cytotoxicity in SW-480 and HT-29 Colon Cancer Cells Through Apoptosis Activation

**Figure 1:**
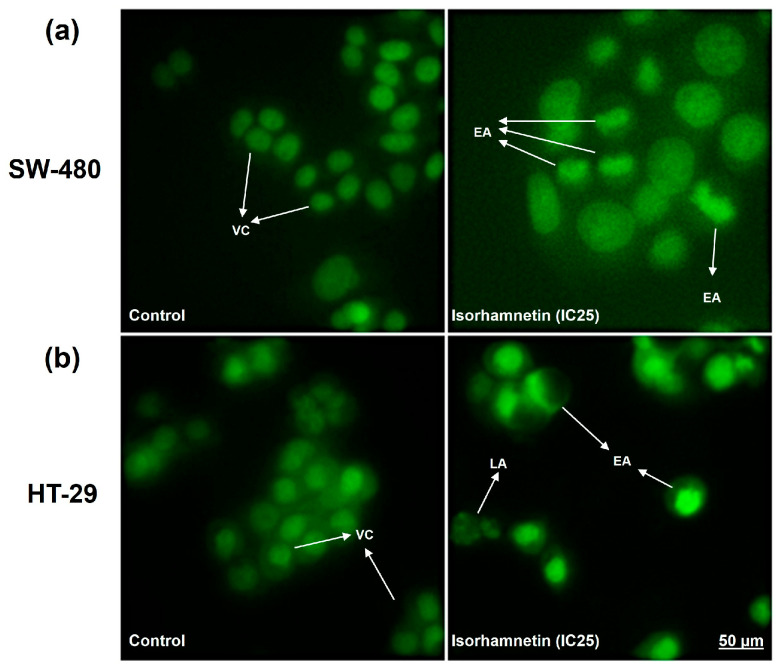
Representative micrographs of SW-480 (**a**) and HT-29 (**b**) control cells and cells with typical apoptotic morphological changes 24 h after treatment with isorhamnetin (IC25 value). VC—viable cells; EA—early apoptosis; LA—late apoptosis.

The obtained micrographs of the treated SW-480 and HT-29 cells (Figure 1) show apoptosis characteristics regarding changes in cell morphology and fluorescent signal, such as a brighter green, fluorescent nucleus with condensed chromatin (early apoptosis, EA) and a dark green, fluorescent nucleus with fragmented chromatin (late apoptosis, LA).

Table 2 presents the percentages of different cell death types, showing that isorhamnetin predominantly induced apoptosis in SW-480 and HT-29 cells 24 h after treatment, while necrosis was minimal, accounting for less than 1% of total cell death.

**Figure 2:**
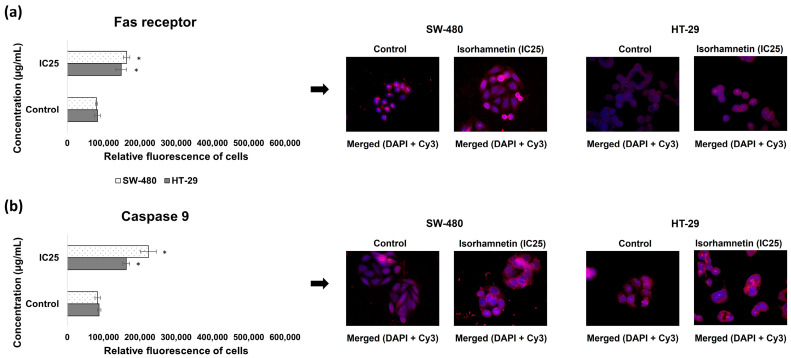
The protein expressions of the Fas receptor (**a**) and Caspase 9 (**b**) in SW-480 and HT-29 colon cancer cells under the influence of isorhamnetin (IC25 value), 24 h after treatment. Cells were visualized using an inverted fluorescent microscope at 600× magnification. The cell nuclei are stained blue (DAPI color), while Fas receptor and Caspase 9 are stained red (secondary antibody conjugated to Cy3). The relative intensity of fluorescence in all cells was measured using the ImageJ program; * *p* < 0.05 compared to untreated cells.

Furthermore, the obtained results demonstrated that isorhamnetin significantly increased the protein level of the Fas receptor, an important death receptor that initiates the extrinsic apoptotic pathway upon ligand binding, in the treated SW-480 and HT-29 cells compared to the controls (Figure 2a). The protein expression of Caspase 9, an initiator caspase in the intrinsic apoptotic pathway, was also elevated under the influence of isorhamnetin in both colon cancer cell lines (Figure 2b). Thus, these results indicate activation of both apoptotic signaling pathways in SW-480 and HT-29 cells following isorhamnetin treatment.

### 2.2. Isorhamnetin Affects the Expression of Drug-Resistance-Related Biomarkers in SW-480 and HT-29 Colon Cancer Cells

As the biotransformation and drug efflux are closely associated with cancer cell resistance development [6,7,8], the effects of isorhamnetin on biomarkers related to these processes were evaluated in SW-480 and HT-29 cells.

#### 2.2.1. Isorhamnetin Reduces the mRNA Expression of CYP Enzymes

**Figure 3:**
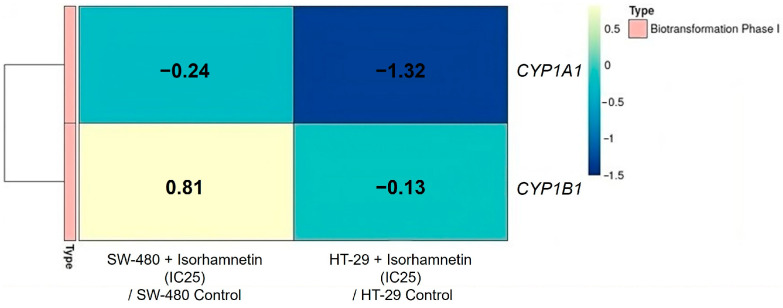
The mRNA expression of phase I biotransformation enzymes in SW-480 and HT-29 cells. The heatmap shows the log2 FC of the target genes between the cells treated with isorhamnetin (IC25 value) and the untreated cells. The color code represents fold change levels: blue indicates downregulation (negative log2 FC values), while yellow indicates overexpression (positive log2 FC values). FC—fold change.

The obtained results (Figure 3) (Table S2) showed that isorhamnetin affects the mRNA expression of target CYP enzymes involved in phase I biotransformation. In the treated SW-480 cells, isorhamnetin significantly reduced only the *CYP1A1* mRNA level compared to untreated controls. Also, in HT-29 cells, the mRNA expression of *CYP1A1* was significantly reduced, while *CYP1B1* showed a decreasing trend compared to the controls.

#### 2.2.2. Isorhamnetin Increases *GSTP1* mRNA Expression and Glutathione Synthesis

**Figure 4:**
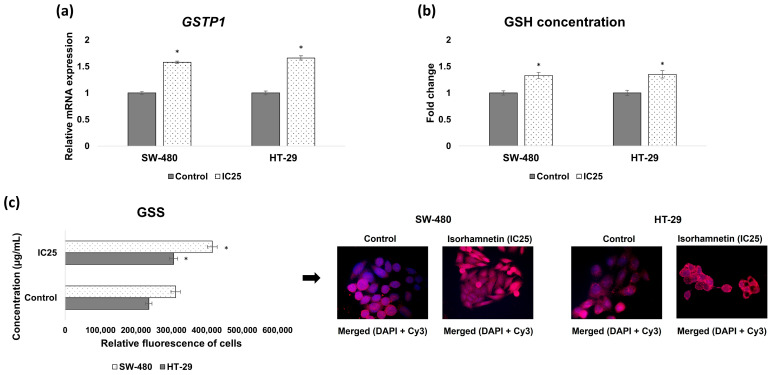
Influence of isorhamnetin on the phase II biotransformation in SW-480 and HT-29 cells, 24 h after applied treatment (IC25 value): (**a**) the mRNA expression of *GSTP1*; (**b**) the concentration of GSH; (**c**) the protein expression of GSS. Cells were visualized using an inverted fluorescent microscope at 600× magnification. The cell nuclei are stained blue (DAPI color), while GSS is stained red (secondary antibody conjugated to Cy3). The relative intensity of fluorescence in all cells was measured using the ImageJ program; * *p* < 0.05 compared to untreated cells. GSH—reduced glutathione; GSS—glutathione synthetase.

According to the results presented in Figure 4a,b, isorhamnetin induced a significant increase in *GSTP1* mRNA expression and concentration of reduced glutathione (GSH) in both SW-480 and HT-29 cells compared to untreated cells. Furthermore, the protein expression of glutathione synthetase (GSS), a key enzyme in glutathione biosynthesis, was significantly increased in the treated colon cancer cells compared to the controls, considering the quantified fluorescence on the obtained micrographs (Figure 4c).

#### 2.2.3. Isorhamnetin Alters Expression of ABC Transporters on the Transcriptional and Protein Levels

**Figure 5:**
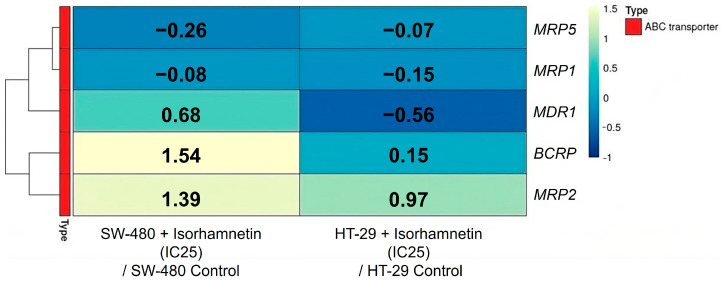
The mRNA expression of ABC transporters in SW-480 and HT-29 cells. The heatmap shows the log2 FC of the target genes between the cells treated with isorhamnetin (IC25 value) and the untreated cells. The color code represents fold change levels: blue indicates downregulation (negative log2 FC values), while yellow indicates overexpression (positive log2 FC values). ABC transporters—ATP-binding cassette transporters; FC—fold change.

**Figure 6:**
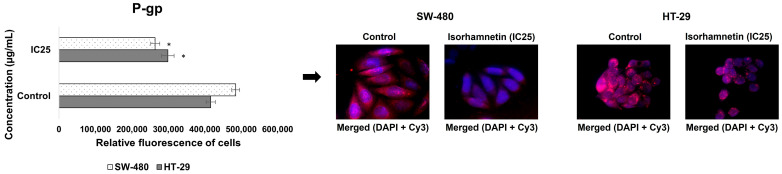
The protein expression of P-gp in SW-480 and HT-29 control cells and cells treated with isorhamnetin (IC25 value), 24 h after treatment. Cells were visualized using an inverted fluorescent microscope at 600× magnification. The cell nuclei are stained blue (DAPI color), while P-gp is stained red (secondary antibody conjugated to Cy3). The relative intensity of fluorescence in all cells was measured using the ImageJ program. * *p* < 0.05 compared to untreated cells. P-gp—P-glycoprotein.

The results (Figure 5) (Table S3) showed that the mRNA levels of *MRP1* and *MRP5* in SW-480 and HT-29 cells had a decreasing trend under the influence of isorhamnetin, while the mRNA levels of *BCRP* and *MRP2* were increased. However, we observed a different mRNA expression of P-gp at the mRNA level (*MDR1* gene) in the treated SW-480 and HT-29 cells compared to the controls. It was increased in SW-480 cells, while in HT-29 cells it was reduced. Nevertheless, the obtained micrographs and measured fluorescence (Figure 6) clearly show that isorhamnetin reduced the protein expression of P-gp in SW-480 and HT-29 cells compared to the controls.

### 2.3. Enhanced Binding Affinity of Isorhamnetin over 5-FU in Molecular Docking with Target Proteins

Molecular docking simulations were used to estimate the molecular interactions between amino acids in active binding sites of the investigated proteins and isorhamnetin and 5-FU. The docking parameters, including free energy of binding (ΔG_bind_), inhibition constant (Ki), and ligand efficiency (LE), are summarized in Table 3, providing quantitative insights into the binding affinity and efficiency of each ligand–protein complex. Lower Ki and more negative ΔG_bind_ values indicate that the investigated ligands interact more effectively with the target proteins.

**Figure 7:**
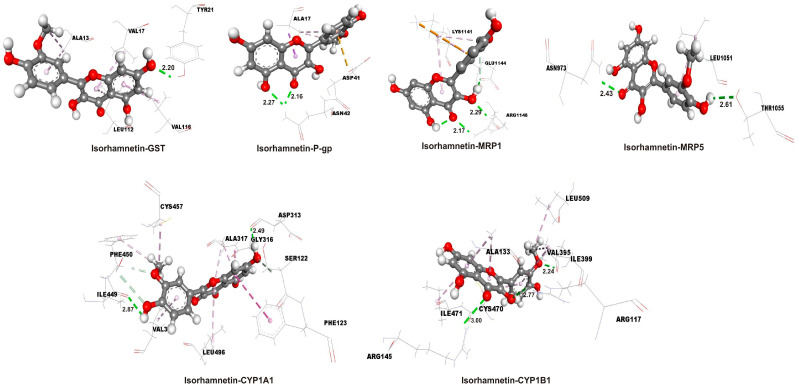
The hydrogen bond (green dotted lines), hydrophobic (rose pink dotted lines), and electrostatic (orange dotted lines) docking interactions of the most stable conformations of the isorhamnetin with target proteins. Distances of the hydrogen bonds are in angstroms, Å. GST—glutathione S-transferase; MRP1—multidrug resistance-associated protein 1; MRP5—multidrug resistance-associated protein 5; P-gp—P-glycoprotein.

**Figure 8:**
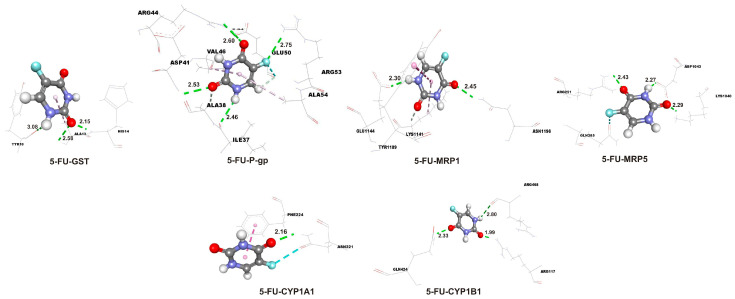
The hydrogen bond (green dotted lines) and hydrophobic (rose pink dotted lines) docking interactions of the most stable conformations of the 5-FU with target proteins. Distances of the hydrogen bonds are in angstroms, Å. 5-FU—5-fluorouracil; GST—glutathione S-transferase; MRP1—multidrug resistance-associated protein 1; MRP5—multidrug resistance-associated protein 5; P-gp—P-glycoprotein.

The results indicate that isorhamnetin consistently exhibits more favorable binding energies (more negative ΔG_bind_) and lower Ki compared to 5-FU across all target proteins. Notably, isorhamnetin demonstrates the strongest binding affinity with CYP1A1 (ΔG_bind_ = −9.0 kcal/mol; Ki = 0.25 µM) and CYP1B1 (ΔG_bind_ = −8.5 kcal/mol; Ki = 0.59 µM), while 5-FU shows its best binding with CYP1A1 (ΔG_bind_ = −5.3 kcal/mol; Ki = 130 µM) and CYP1B1 (ΔG_bind_ = −4.7 kcal/mol; Ki = 360 µM), though these values are less favorable than those of isorhamnetin. The molecular interactions between the ligands and the proteins were analyzed using docking interaction diagrams (Figure 7 for isorhamnetin and Figure 8 for 5-FU). These figures illustrate the hydrogen bonds (green dotted lines), hydrophobic interactions (rose pink dotted lines), and electrostatic interactions (orange dotted lines) between the ligands and amino acid residues in the protein binding sites. Isorhamnetin generally forms more hydrogen bonds compared to 5-FU, particularly with CYP1A1 and CYP1B1, which correlates with its stronger binding affinities as evidenced by more negative ΔG_bind_ values (e.g., −9.0 kcal/mol for isorhamnetin-CYP1A1 vs. −5.3 kcal/mol for 5-FU-CYP1A1, as per Table 3). For GST, 5-FU forms five hydrogen bonds, slightly more than isorhamnetin’s four, but the binding energy still favors isorhamnetin (−5.4 kcal/mol vs. −4.5 kcal/mol), suggesting other interactions like hydrophobic contacts play a role. Hydrogen bond distances typically range from 2.0 to 3.0 Å, with shorter distances indicating stronger interactions. Isorhamnetin has several short bonds, such as TYR21 and ASN42 at 2.16 and 2.20 Å with GST. 5-FU also has short bonds, like PHE224 at 2.16 Å with CYP1A1 and ARG117 at 1.99 Å with CYP1B1, but fewer in number. Notably, ASP41 in 5-FU-GST at 3.08 Å is at the upper limit, suggesting a weaker hydrogen bond. Isorhamnetin interacts with a diverse set of residues, including polar (SER122 and THR1055), charged (ASP313, LYS1141, and ARG1148), and hydrophobic (LEU496, VAL3, and PHE450). For example, with CYP1A1, it forms bonds with SER122 and ASP313, enhancing polar interactions. 5-FU also interacts with polar and charged residues, such as ARG, GLN, and ASN, but its interactions are more limited, especially with CYP enzymes. The interaction profiles correlate with the docking parameters provided in Table 3, where isorhamnetin consistently shows lower Ki and more negative ΔG_bind_ compared to 5-FU. For example, isorhamnetin’s Ki for CYP1A1 is 0.25 µM (ΔG_bind_ = −9.0 kcal/mol) versus 130 µM for 5-FU (ΔG_bind_ = −5.3 kcal/mol), indicating a significantly stronger binding. This is likely due to isorhamnetin’s ability to form multiple interaction types, including more hydrogen bonds and hydrophobic contacts, as observed in Figure 7 and Figure 8. The summary of interactions highlights isorhamnetin’s superior binding affinity to CYP1A1, CYP1B1, GST, P-gp, MRP1, and MRP5 compared to 5-FU, driven by more hydrogen bonds, extensive hydrophobic interactions, and potential electrostatic contributions.

## 3. Discussion

Colon cancer has been one of the most dominant diseases in recent years. Since this is a multifactorial disease that develops very slowly, diagnosis in the early stages is difficult. Several well-known therapeutic strategies are in clinical use to struggle against the uncontrolled proliferation and invasion of these cells into surrounding tissues, intending to improve the patient’s health [31]. However, the high cost and the development of resistance to commercial cytostatics, as well as their numerous side toxic effects, have directed research toward the investigation of natural products that are used in traditional medicine.

Natural products derived from plants, as the most important natural source, classified as alkaloids, terpenoids, and phenolic compounds (phenols, flavonoids, phenolic acids, etc.) have shown many anticancer properties [32]. In fact, these compounds make up a significant part of anticancer drugs in clinical use. They are used in the form obtained from plants or as semi-synthetic chemicals by modifying their primary structure, as is the case with paclitaxel and its derivative docetaxel [33].

Since quercetin has shown favorable results as an anticancer agent against colon cancer by reducing the cell viability of HT-29, HCT-15 [34], Caco-2, SW-620 [35], and HCT-116 immortalized as well as primary COLO 320 and metastatic COLO 205 colon cancer cell lines [36], the focus in this paper was to analyze its metabolite, isorhamnetin. Our results showed that isorhamnetin significantly decreases the viability of SW-480 and HT-29 colon cancer cells, indicating its antiproliferative effect. In this regard, the obtained IC50 values, as a measure of cytotoxicity, suggest that this compound exhibits high cytotoxicity against SW-480 and HT-29 cells according to National Cancer Institute (NCI) criteria (IC50 ≤ 20 μg/mL) [37]. These findings are consistent with previous reports showing similar cytotoxic effects against HT-29 and Caco-2 [23], while a somewhat weaker effect was observed in HCT-116 cells [22]. In our previous study [38], 5-FU demonstrated higher IC50 values on these colon cancer cell lines (values for 24 h, 30.59 and 32.87 µg/mL, and values for 72 h, 2.20 and 2.69 µg/mL, for SW-480 and HT-29 cells, respectively), suggesting that isorhamnetin may have stronger cytotoxic potential. Compared to other cancer cell types, such as BEL-7402 hepatocellular carcinoma cells (IC50, 74.4 µg/mL, and 72 h) [26] and T24 (IC50, 127.86 µM, and 48 h) and T5637 (IC50, 145.75 µM, and 48 h) bladder cell lines [25], isorhamnetin demonstrated considerably stronger cytotoxicity against colon cancer cells. Notably, the cytotoxic effect on SW-480 cells appeared to weaken after 72 h of exposure, as indicated by increased IC50 values, suggesting a potential reduction in sensitivity upon extended treatment. This observation underscores the importance of further studies to investigate the long-term effects of isorhamnetin on colon cancer cells, particularly to determine whether prolonged exposure may lead to the development of cellular resistance. Such insights will be critical for evaluating the therapeutic potential of isorhamnetin. Therefore, the 24 h time point was selected as the primary time point for most experiments, as it provides relevant insight into specific cellular responses and gene expression changes that can be measured without significant cell death or secondary effects. This timeframe enables the detection of both immediate early gene responses and downstream transcriptional changes, offering a comprehensive overview of the treatment’s effects.

Furthermore, isorhamnetin exhibited selective cytotoxicity against both colon cancer cell lines, with a more pronounced effect on SW-480 cells compared to HT-29 cells, as indicated by the SI values. This observation aligns with previously published data, including the study by Antunes-Ricardo and colleagues [23], who reported similar sensitivity of normal NIH 3 T3 fibroblasts to isorhamnetin (IC50, 30.3 µg/mL, and 48 h), consistent with our observations on normal HaCaT keratinocytes. Importantly, isorhamnetin-induced cytotoxicity was primarily mediated through apoptosis, as evidenced by characteristic apoptotic cell morphological changes and the significant upregulation of Fas receptor and Caspase 9 protein levels. These findings suggest the activation of both extrinsic and intrinsic apoptotic pathways in colon cancer cells following isorhamnetin treatment. Our results are in line with previous reports on colon cancer cells [22,23] and are further supported by studies in breast [24], bladder [25], and melanoma [27] cancer cells. Collectively, these findings reinforce the notion that isorhamnetin possesses potent proapoptotic activity and highlight its promising anticancer potential, further supported by its favorable pharmacokinetic profile, notably its prolonged plasma presence compared to quercetin, as reported by Park and coworkers [39].

In addition to numerous anticancer properties, another important advantage of natural compounds is their influence on parameters related to the development of drug resistance in cancer cells. In this regard, plant-derived compounds have found a special place, as they are classified as fourth-generation inhibitors of ABC transporters [15,18]. Enhanced activity of these pumps leads to the efflux of cytostatics from the cancer cells and represents a major mechanism of drug resistance development [6,7,8]. However, many ABC transporters require previously metabolized drugs by CYP enzymes and conjugated with glutathione by GSTs to exert their function [12,40]. The CYP1A1 and CYP1B1 enzymes play an important role in xenobiotic metabolism and the biotransformation of various plant-derived compounds and chemotherapeutic agents [41]. Although many other CYP isoforms contribute to these processes and their analysis could provide additional valuable insights, we focused on CYP1A1 and CYP1B1 because these isoforms are frequently overexpressed in colon cancer cells [42]. Moreover, the interaction of isorhamnetin with these isoforms has not yet been thoroughly investigated in these cells. Our results indicate that isorhamnetin reduced the mRNA expression of *CYP1A1* in SW-480 and HT-29 cells, while the *CYP1B1* mRNA level showed a decreasing trend only in the treated HT-29 cells. These findings contrast with reports showing that quercetin is an effective inducer of *CYP1* (*A1*, *A2*, and *B1*) mRNA expression, whereas kaempferol, which differs in one hydroxyl group, does not affect the mRNA expression of these genes in Caco-2 colon cancer cells [43]. Notably, these results also differ from our previous study [38], where 5-FU increased the mRNA expression of *CYP1A1* and *CYP1B1* in the same colon cancer cell lines. This suggests that isorhamnetin may differentially modulate the expression of target CYP enzymes, which may lead to distinct effects on drug metabolism. By downregulating *CYP1A1* and *CYP1B1* enzymes, isorhamnetin treatment could potentially improve the efficacy of cytostatics by reducing their metabolism and clearance, thereby enhancing their therapeutic activity; however, this hypothesis requires future validation. Conversely, our findings showed that isorhamnetin significantly increased *GSTP1* mRNA expression and GSH concentrations in both SW-480 and HT-29 cells. Moreover, the protein expression of GSS, an enzyme involved in de novo glutathione synthesis, was markedly elevated in the treated colon cancer cells. These results indicate activation of cellular detoxification pathways, which could represent an adaptive response to isorhamnetin-induced cytotoxic stress. Given that *GSTP1* is highly expressed in colon cancer cells and that elevated GSH levels can influence the efficacy of cytostatics [44], the increase in GSH and activation of detoxification pathways may support cellular defense mechanisms. However, it is important to note that the passage of isorhamnetin through cellular detoxification systems does not necessarily result in its inactivation or reduced potency. Despite the engagement of these pathways, our findings indicate that isorhamnetin nevertheless maintains significant cytotoxic and proapoptotic effects in colon cancer cells. Molecular docking analysis revealed a very high binding affinity of isorhamnetin for the active site of GST, as well as for CYP1A1 and CYP1B1, strongly suggesting a direct interaction with these proteins. These findings are particularly significant, as they suggest that isorhamnetin may compete with cytostatics for the GST binding site, potentially weakening their binding to GST and reducing their efflux from the cells. Similarly, the strong binding affinity of isorhamnetin for CYP1A1 and CYP1B1 indicates that it could interfere with the metabolism of cytostatics by these enzymes, potentially modulating their therapeutic efficacy. Further studies are warranted to confirm these interactions and fully elucidate their implications for isorhamnetin’s therapeutic potential.

Although various mechanisms contribute to the development of drug resistance in cancer cells, the most significant one, which was the focus of our research, involves ABC transporters. There is a lack in the literature on the potential of isorhamnetin to inhibit the mRNA expression of key ABC transporters. The P-gp, MRP1, MRP2, MRP5, and BCRP are known to be expressed in colon cancer cells, as reported by other authors [45,46], which provided the rationale for selecting these ABC transporters for our study. Our results indicate that isorhamnetin has the potential to suppress *MRP1* and *MRP5* mRNA expression in SW-480 and HT-29 cells. Conversely, the observed increase in *MRP2* mRNA expression, which serves as an export pump for glutathione conjugates, together with increased GSH levels, GSS protein expression, and *GSTP1* mRNA expression, could indicate that isorhamnetin is being exported from treated SW-480 and HT-29 cells in the form of GSH-conjugates. Interestingly, the inhibitory effect of isorhamnetin on P-gp mRNA expression (*MDR1* gene), the major ABC transporter closely related to drug resistance development [6,7,8], differed between these cell lines. Inhibition at the mRNA level was observed only in the HT-29 cells. However, mRNA levels alone are insufficient to predict protein levels due to posttranscriptional regulation [47]. Importantly, we demonstrated that isorhamnetin significantly decreased the expression of P-gp at the protein level in both SW-480 and HT-29 cells. In addition, molecular docking analysis revealed strong interactions between isorhamnetin and the binding site of P-gp, further supporting its potential to modulate the activity of this transporter. In contrast, our previous study [38] showed that 5-FU significantly increased the mRNA expression of all investigated ABC transporters, as well as P-gp protein levels, in these colon cancer cells, underscoring a distinct regulatory profile for isorhamnetin with potential therapeutic advantages. These results indicate that isorhamnetin should be considered as another phytochemical with the potential to interfere with P-gp expression, similar to other plant-derived compounds (glaucine, piperine, genistein, kaempferol, curcumin, quercetin, etc.) [17,18,19]. Future in vitro and in vivo studies are necessary to confirm whether isorhamnetin could enhance the efficacy of cytostatics used in colon cancer therapy by reducing their efflux from cells, especially those that are substrates for P-gp. These findings could contribute to the development of novel therapeutic strategies for colon cancer, with a focus on preventing the development of drug resistance.

Furthermore, the docking study reliably demonstrated that isorhamnetin effects could be mediated through several, possibly simultaneously, affected protein targets. Ganbold and colleagues [48] suggested that methyl groups at the C3’ position of isorhamnetin may have functional roles in exerting its biological activity. This suggests a specific mode of interaction between isorhamnetin and target proteins, which could be crucial for understanding isorhamnetin’s biological activity and potential therapeutic effects. Further exploration of these interactions could shed light on the mechanisms underlying the pharmacological effects of isorhamnetin, leading to the development of novel therapies targeting CYP enzymes and ABC transporters. These in silico findings, together with great effects on SW-480 and HT-29 colon cancer cells, provide a foundation for further experimental validation and characterization of isorhamnetin’s interactions with target proteins, including CYP1A1, CYP1B1, P-gp, MRP1, and MRP5. If corroborated by subsequent in vivo studies, these results could support the development of isorhamnetin-based therapies targeting diseases where these target proteins and related proteins play significant roles, such as the development of drug resistance. Overall, these in silico and in vitro findings underscore the potential pharmacological significance of isorhamnetin and warrant further investigation both on other cell types and animal models to validate its therapeutic potential and mechanism of action.

## 4. Materials and Methods

### 4.1. Chemicals

Dulbecco’s Modified Eagle Medium (DMEM), phosphate-buffered saline (PBS), 0.25% trypsin-EDTA, and penicillin/streptomycin were bought from Capricorn Scientific GmbH, Ebsdorfergrund, Germany. Fetal bovine serum (FBS) was obtained from PAN Biotech, Aidenbach, Germany. Acridine Orange (AO) was from Acros Organics, Fair Lawn, NJ, USA, while ethidium bromide (EB), Dimethyl sulfoxide (DMSO), and 3-[4,5-dimethylthiazol-2-yl]-2,5-diphenyltetrazolium bromide (MTT) were obtained from SERVA, Heidelberg, Germany. RNA Extracol, a reverse transcription kit (NG dART RT kit), and a qPCR Master Mix (SG/ROX qPCR Master Mix) were purchased from EURx, Gdańsk, Poland. Primers were synthesized by Microsynth, Balgach, Switzerland. The primary antibodies Fas and Caspase 9 were from R&D Systems, Minneapolis, MN, USA, while the primary antibody GSS was obtained from Sigma Aldrich, St. Louis, MO, USA. The diamidino-2-phenylindole (DAPI), secondary antibody conjugated with Cy3, and the primary P-gp antibody were bought from Thermo Scientific, Waltham, MA, USA.

### 4.2. Cell Lines

Human colorectal adenocarcinoma cell lines SW-480 and HT-29 were obtained from the American Type Culture Collection, Manassas, VA, USA, while the normal human keratinocytes cell line HaCaT was purchased from Cell Lines Service, Eppelheim, Germany. All cells were cultivated in DMEM containing 10% FBS, 100 U/mL penicillin, and 100 μg/mL streptomycin, at 37 °C in a 5% CO_2_ incubator.

### 4.3. Isorhamnetin

Isorhamnetin was obtained from TCI Europe N.V., Zwijndrecht, Belgium (Cat. No. 480–19-3). The compound was dissolved in DMSO and then diluted in DMEM to a final concentration of 500 μg/mL. The DMSO concentration in the isorhamnetin stock solution was maintained at <1%. Before the experiments, all necessary concentrations were prepared from the stock solution in DMEM, with the DMSO concentration being 0.05% at the highest concentration used.

### 4.4. Cell Viability Assay

An MTT colorimetric assay was performed to examine the impact of isorhamnetin on SW-480, HT-29, and HaCaT cell viability [49]. When the cells reached 80% confluence in 75 cm^2^ culture flasks, they were trypsinized, counted, and seeded in 96-well plates at a density of 10^4^ cells per well. After overnight incubation, DMEM was replaced with 100 μL of an appropriate isorhamnetin concentration (range 0.1–25 μg/mL) for 24 and 72 h. Untreated cells served as a control. The experimental procedure was described in detail by Nikodijević and colleagues [50]. The IC50 was calculated by the CalcuSyn program based on the dose–response curves. The cytotoxic selectivity of isorhamnetin, which is expressed as the SI, was calculated according to the following formula: IC50 of the normal cell line (HaCaT)/IC50 of the cancer cell line (SW-480 and HT-29).

### 4.5. Cell Death Analysis by Fluorescent Staining

To analyze the type of cell death of SW-480 and HT-29 cells, an Acridine Orange/ethidium bromide (AO/EB) double-staining assay was used [51]. Cells (10^4^ cells/well) were treated for 24 h with isorhamnetin at a concentration of IC25 (determined for each cell line using the MTT assay), while untreated cells served as controls. When the treatment expired, 10 μL of AO and 10 μL of EB dye (100 μg of dye per 1 mL of distilled water) were added to each sample. At least 300 cells were counted per sample according to the dye and nuclei shape using an inverted fluorescent microscope (Nikon Ti-Eclipse, Nikon Corporation, Tokyo, Japan) at 400× magnification. The percentages of viable (VC), EA, LA, and necrotic cells (NC) were calculated concerning the total cell number for every sample.

### 4.6. Concentration of Reduced Glutathione

A colorimetric method [52] was performed to determine the concentration of GSH. After seeding the SW-480 and HT-29 cells in 96-well plates (5 × 10^4^ cells/well) and at the end of the pre-incubation period, they were treated with isorhamnetin at the IC25 concentration obtained by the MTT assay for each cell line. Untreated cells served as controls. Further procedure, previously described in detail by Nikodijević and colleagues [50], was performed 24 h after treatment. The results were first expressed in nmol/mL concerning a standard curve formed by known molar concentrations of BSA and then calculated in relation to the number of viable cells (according to the results of the MTT assay). Finally, the results were presented as a fold change in GSH concentration in the treated SW-480 and HT-29 cells, relative to the control cells.

### 4.7. RNA Isolation, Reverse Transcription, and Relative mRNA Quantification

The SW-480 and HT-29 cells were seeded in 25 cm^2^ culture flasks (10^6^ cells/flask) and treated with an isorhamnetin IC25 concentration (observed by MTT assay for both cell lines) at 80% confluence. In control flasks, only DMEM was replaced. Isolation of total RNA was performed 24 h after treatment by the phenol–chloroform method [53] using RNA Extracol. The Eppendorf BioPhotometer Plus (Eppendorf AG, Hamburg, Germany) was used to measure the RNA concentration in all samples. All samples were aliquoted to a final concentration of 1 μg/μL.

For the transcription of RNA into complementary DNA (cDNA), the commercial NG dART RT kit was used. At each sample, 4 μL of 5 × NG cDNA buffer, 1 μL of random hexamer primer, 2 μL RNA of samples, 1 μL NG dART RT MIX, and 12 μL RNase-free water were added. The samples were heated in the Eppendorf Mastercycler PCR by the following temperature conditions: 25 °C for 10 min, 50 °C for 50 min, and 85 °C for 5 min.

Relative mRNA quantification of the analyzed genes was performed by SG/ROX qPCR Master Mix. The PCR mixture was optimized for a 20 μL reaction. It contained 10 μL of the SG/ROX qPCR Master Mix (2×), 0.5 μL of forward and 0.5 μL of reverse primer (10 μM) for the target gene, 1 μL cDNA of samples, and 8 μL nuclease-free water per sample. Amplification and quantification of PCR products were performed using the Applied Biosystems, Quantitative Real-Time system (Applied Biosystems 7500, Real-Time PCR Software v2.0; Applied Biosystems, Foster City, CA, USA) by the following thermal cycling conditions: 10 min of initial denaturation at 95 °C, followed by 40 cycles of 94 °C for 15 s, 60 °C for 30 s, and 72 °C for 30 s. Melt curve analysis was performed between 60 and 94 °C. β-actin was used as an internal control to normalize the mRNA. The obtained results were analyzed by the 2^−ΔΔCt^ method [54]. Primer sequences for each gene are presented in Table S1.

### 4.8. Immunofluorescence

The Fas receptor, Caspase 9, GSS, and P-gp protein expressions were determined using immunofluorescence staining [55]. The SW-480 and HT-29 cells were seeded on glass coverslips in 6-well plates (5 × 10^5^ cells/well). After reaching 80% confluence, the medium was removed, and the cells were treated with isorhamnetin at an IC25 concentration (determined by the MTT assay for both cell lines) for 24 h. In the control cells, DMEM was replaced. At the end of the treatment period, further fluorescence staining procedures were performed as described by Nikodijević and colleagues [50]. Micrographs were captured using an inverted fluorescent microscope (Nikon Ti-Eclipse) at 600× magnification. Quantification of cell fluorescence on the obtained micrographs was performed by ImageJ software version 1.51k (Wayne Rasband, ImageJ, https://imagej.net/ij/, accessed on 5 May 2025), with results presented as relative fluorescence per cell.

### 4.9. Statistical Analysis

All analyses were carried out in the three individual experiments in triplicate for each dose. The data are expressed as mean ± standard error (SE). Statistical analysis was performed using the SPSS statistical software package (IBM SPSS Statistics Version 23, 2015). An independent *t*-test and one-way ANOVA test for multiple comparisons followed by a Tukey HSD post hoc test were used to assess significant differences between treated and control cells. Statistical significance was set at *p* < 0.05.

Heatmaps were created online in ClustVist: a web tool for visualizing the clustering of multivariate data (BETA) (https://biit.cs.ut.ee/clustvis/, accessed on 10 May 2025). The data were clustered using the maximum clustering method for the rows, while the tree order for the rows was the lower mean value first.

### 4.10. Molecular Docking

AlphaFold is an Artificial Intelligence (AI) system developed that predicts the 3D structure of a protein from its amino acid sequence [56]. AlphaFold has proved to be of great value for AI in chemistry, biology, and medicine. The AlphaFold algorithm is based on a deep neural network. This method combines the characteristics of proteins derived from homologous templates and from multiple sequence comparisons to generate the predicted structure. This approach has shown remarkable accuracy in an otherwise unknown structural set. In this paper, AlphaFold was used to obtain high-quality models of target proteins: CYP1A1 (code: P04798), CYP1B1 (code: Q16678), and GST (code: Q5K5Z8) and ABC transporters: P-gp (code: F4ZUQ5), MRP1 (code: P33527), and MRP5 (code: O15440). Before docking computations, the protonation state (physiological pH, pH = 7.4) of the target proteins was examined using AMDock software version 1.5.2. The predicted protonation states were used to refine the docking poses to improve the accuracy of the protein–ligand interactions.

Molecular docking simulations were used to assess the isorhamnetin’s potential interaction with the investigated proteins. The 5-FU was also tested for its ability to interact with the investigated proteins. The AutoDock Vina software version 1.2.1 [57] implemented in the AMDock program [58] was used to examine the binding affinity of the investigated compounds against proteins. The protein pockets and binding sites were determined using the AMDock program. The box centers with dimensions −1.1 × 1.9 × 1.3 Å^3^ of CYP1A1; −2.2 × −1.9 × 5.8 of CYP1B1 Å^3^; −3.4 × −0.9 × 3.4 Å^3^ of GST; −1.1 × 1.7 × 3.3 Å^3^ of P-gp; −4.0 × 3.3 × −2.0 Å^3^ of MRP1; and −10.6 × 2.1 × −3.0 Å^3^ of MRP5 in the -x, -y, and -z directions were employed to cover the protein binding site and allow the ligand to move freely. The title molecules’ binding affinity was investigated and discussed. The free energy of the binding values is calculated by the AutoDock Vina software using the following equation, Equation (1):

(1) $$Ki=e^{\frac{\Delta G_{\mathrm{bind}}}{\mathrm{RT}}}$$

where Ki is the inhibition constant, ΔG_bind_ is the binding free energy, R is the universal gas constant, and T is the temperature. Ligand efficiency (LE) represents the binding energy of the ligand to protein per atom. LE (Equation (2)) has a unit of kJ/mol/heavy atom.

(2) $$\mathrm{LE}=\frac{\Delta G_{\mathrm{bind}}}{N}$$

## 5. Conclusions

Isorhamnetin exhibits strong cytotoxic and proapoptotic effects against SW-480 and HT-29 colon cancer cells, confirming its potential in colon cancer therapy. This study further expands current knowledge by highlighting isorhamnetin’s ability to modulate biotransformation enzymes and drug efflux transporters involved in drug resistance in colon cancer cells, notably through the suppression of P-gp protein expression and reduction in *CYP1A1*, *CYP1B1*, *MRP1,* and *MRP5* mRNA levels. Molecular docking analysis revealed a high binding affinity of isorhamnetin not only to CYP1A1, CYP1B1, and GST but also to key drug-resistance-associated transporters, including P-gp, MRP1, and MRP5, exceeding that of 5-FU, suggesting its potential to interfere with their function directly. Although these results are promising, the study is limited by its in vitro nature and the need for in vivo validation to confirm therapeutic efficacy and safety. Overall, isorhamnetin remains a promising candidate for modulating drug resistance in colon cancer cells, deserving further investigation.

## Figures and Tables

**Table 1:** The cytotoxic effect of isorhamnetin on HaCaT, SW-480, and HT-29 cells expressed as the IC50 value (µg/mL) and selectivity index (SI) values.

	Isorhamnetin			
	IC50 Value (µg/mL)		SI	
Cell line	24 h	72 h	24 h	72 h
HaCaT	24.70 ± 1.89	23.33 ± 0.42		
SW-480	1.59 ± 0.09	2.09 ± 0.09	15.54	11.16
HT-29	26.07 ± 0.34	8.65 ± 0.20	0.95	2.69

**Table 2:** The percentages of viable, early, and late apoptotic and necrotic SW-480 and HT-29 cells, 24 h after treatment with isorhamnetin.

		Isorhamnetin			
Cell Line	µg/mL	Viable Cells	Early Apoptosis	Late Apoptosis	Necrosis
SW-480	0	92.92 ± 0.07	5.88 ± 0.31	1.08 ± 0.38	0.12 ± 0.12
	IC25	80.36 ± 1.44 *	15.69 ± 1.12 *	3.86 ± 0.52 *	0.09 ± 0.09
HT-29	0	96.42 ± 0.44	3.23 ± 0.61	0.35 ± 0.17	0
	IC25	66.13 ± 0.95 *	24.32 ± 2.86 *	9.10 ± 2.94 *	0.45 ± 0.45

* *p* < 0.05 compared to untreated cells.

**Table 3:** The important parameters for the best docking conformations of isorhamnetin and 5-FU with protein targets, the free energy of binding (ΔG_bind_), inhibition constant (Ki), and ligand efficiency.

Complex	ΔG_bind_ (kcal mol^−1^)	Ki (µM)	LE
Isorhamnetin-CYP1 A1	−9.0	2.50 × 10^−^^1^	−0.39
Isorhamnetin-CYP1B1	−8.5	5.90 × 10^−^^1^	−0.37
Isorhamnetin-GST	−5.4	1.10 × 10^2^	−0.23
Isorhamnetin-P-gp	−6.5	1.72 × 10^1^	−0.28
Isorhamnetin-MRP1	−5.8	5.61 × 10^1^	−0.25
Isorhamnetin-MRP5	−6.7	1.23 × 10^1^	−0.29
5-FU-CYP1A1	−5.3	1.30 × 10^2^	−0.59
5-FU-CYP1B1	−4.7	3.60 × 10^2^	−0.52
5-FU-GST	−4.5	5.00 × 10^2^	−0.50
5-FU-P-gp	−4.3	7.00 × 10^2^	−0.48
5-FU-MRP1	−3.7	1.94 × 10^3^	−0.41
5-FU-MRP5	−4.1	9.90 × 10^2^	−0.46

## Data Availability

Data will be available on demand.

## Supplementary Materials

The following supporting information can be downloaded at https://www.mdpi.com/article/10.3390/ijms26136208/s1.

## Abbreviations

The following abbreviations are used in this manuscript:

5-FU	5-fluorouracil
ABC transporters	ATP Binding Cassette (ABC) transporters
AI	Artificial Intelligence
AO	Acridine Orange
BCRP	Breast cancer resistance protein
BSA	Bovine serum albumin
cDNA	Complementary DNA
CYP	Cytochrome P450
DAPI	Diamidino-2-phenylindole
DMEM	Dulbecco’s Modified Eagle Medium
DMSO	Dimethyl sulfoxide
EA	Early apoptotic cells
EB	Ethidium bromide
FBS	Fetal bovine serum
FC	Fold change
GCO	Global Cancer Observatory
GSH	Reduced glutathione
GSS	Glutathione synthetase
GSTs	Glutathione S-transferases
IC50	The concentration that inhibits 50% of cell growth
LA	Late apoptotic cells
LE	Ligand efficiency
MRP1	Multidrug resistance-associated protein 1
MRP5	Multidrug resistance-associated protein 5
MTT	3-[4,5-dimethylthiazol-2-yl]-2,5-diphenyltetrazolium bromide
NBDs	Nucleotide-binding domains
NC	Necrotic cells
NCI	National Cancer Institute
PBS	Phosphate-buffered saline
P-gp	P-glycoprotein
SE	Standard error
SI	Selectivity index
TMDs	Transmembrane domains
VC	Viable cells
