# Supplementary material for: Isorhamnetin Modulates Drug-Resistance-Related Biomarkers in Colon Cancer Cells
Source: Int J Mol Sci. 2025 Jun 27;26(13):6208. doi: 10.3390/ijms26136208 (PMC12250396; doi:10.3390/ijms26136208)
Supplement: Supplementary file 1 [file ijms-26-06208-s001.zip › ijms-3696024-supplementary.pdf]

Table S1. Primer sequences used for the quantification of mRNA expression

Gene	Forward sequence	Reverse sequence
CYP1A1	*5′-GATTGAGCACTGTCAGGAGAAGC-3′*	*5′-ATGAGGCTCCAGGAGATAGCAG-3′*
CYP1B1	*5′-TGATGGACGCCTTTATCCTCTC-3′*	*5′-CATAAAGGAAGGCCAGGACATA-3′*
GSTP1	*5′-TCAAAGCCTCCTGCCTATAC-3′*	*5′-AGGTGACGCAGGATGGTATT-3′*
MDR1	*5′-GCCTGGCAGCTGGAAGACAAATACACAAAATT-3′*	*5′-CAGACAGCAGCTGACAGTCCAAGAACAGGACT-3′*
BCRP	*5′-TATAGCTCAGATCATTGTCACAGTC-3′*	*5′-GTTGGTCGTCAGGAAGAAGAG-3′*
MRP1	*5′-ACCCTAATCCCTGCCCAGAG-3′*	*5′-CGCATTCCTTCTTCCAGTTC-3′*
MRP2	*5′-GCCAACTTGTGGCTGTGATAGG-3′*	*5′-ATCCAGGACTGCTGTGGGACAT-3′*
MRP5	*5′-ATTTGGACCCCTTCAACCAGTAC-3′*	*5′-GGTAGCTGAGCAATACATTCTTTCAT-3′*
β-actin	*5′-AAGCAGGAGTATGACGAGTCCG-3′*	*5′-GCCTTCATACATCTCAAGTTGG-3′*

Table S2. Expression of mRNA of target genes involved in phase I biotransformation in SW-480 and HT-29 cells under the influence of isorhamnetin (IC25). The result is presented as the fold change mRNA expression in a target sample, relative to a control sample normalized to a reference gene

	Isorhamnetin (μg/mL)		
**Cell line**	Concentration	*CYP1A1*	*CYP1B1*
**SW-480**	0	1	1
	IC25	0.85±0.01*	1.75±0.03*
**HT-29**	0	1	1
	IC25	0.40±0.03*	0.91±0.01*

*p<0.05 compared to untreated cells

Table S3. Expression of mRNA of target ABC transporters involved in drug efflux process in SW-480 and HT-29 cells under the influence of isorhamnetin (IC25). The result is presented as the fold change mRNA expression in a target sample, relative to a control sample normalized to a reference gene

		Isorhamnetin (μg/mL)				
**Cell line**	Concentration	*MDR1*	*BCRP*	*MRP1*	*MRP2*	*MRP5*
**SW-480**	0	1	1	1	1	1
	IC25	1.60±0.04*	2.91±0.06*	0.94±0.01*	2.62±0.05*	0.83±0.02*
**HT-29**	0	1	1	1	1	1
	IC25	0.67±0.025*	1.11±0.02*	0.90±0.01*	1.96±0.03*	0.95±0.015*

*p<0.05 compared to untreated cells

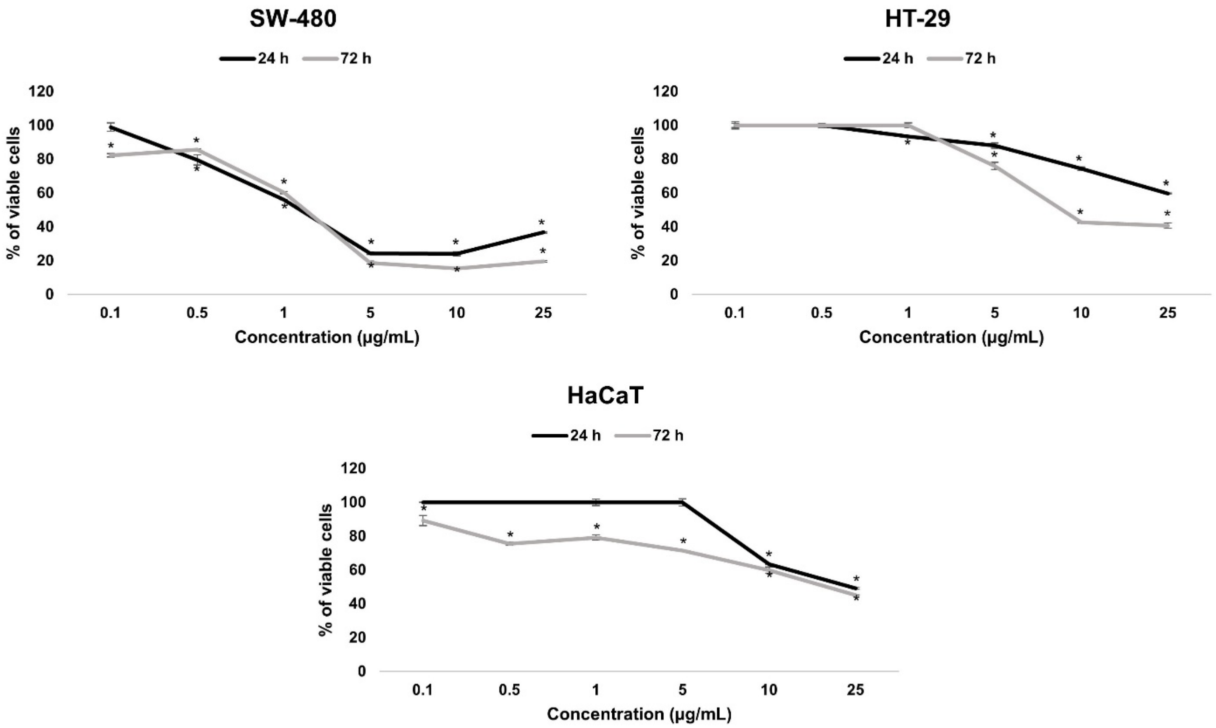

Figure S1. Effect of isorhamnetin on the viability of SW-480 and HT-29 colon cancer cells and normal HaCaT cells. *$p<0.05$ compared to control values
